# COVID-19: Where are the Nigerian social workers?

**DOI:** 10.1177/1473325020973336

**Published:** 2021-03

**Authors:** Stanley Oloji Isangha, Wai Man Anna Choi, Marcus Yu Lung Chiu

**Affiliations:** Department of Social and Behavioral Sciences, College of Liberal Arts and Social Sciences, City University of Hong Kong, Kowloon, Hong Kong SAR

**Keywords:** Psychosocial, poverty, social work practice, Nigeria, COVID-19

## Abstract

The emergence of COVID-19 pandemic has brought untold hardship across the globe. Developed nations have taken relatively commendable actions to quell its impact on livelihood and most have also included social workers in the frontline due to their expertise in working with vulnerable populations. Same cannot be said of developing nations particularly Nigeria who hurriedly copied the measures adopted by the developed nations without carefully considering her peculiarities. Given Nigeria’s high poverty rate prior to and even higher during the pandemic as well as the few available resources, it is important that Nigerian social workers should be called upon as frontline workers with regards to the welfare of the vulnerable and the psychosocial well-being of infected persons and their families. Instead, Nigeria has totally ignored the importance of social workers and palliatives have been stolen by those tasked with distribution while the psychosocial well-being of affected persons has been left to fate.

## Introduction

The emergence of COVID-19, following the outbreak of SARS-CoV in 2002 and MERS-CoV in 2012, has brought developed nations such as the US and the UK to their knees begging for divine intervention to revive socioeconomic activities in their search for the vaccine (Meo et al., 2020; Nassar et al., 2018). This leaves us to wonder what the fate of Africa is, particularly Nigeria, where 82.9 and 150 million people live in extreme poverty and lack access to clean water and/or sanitary services respectively (Aljazeera, 2020; Wateraid, 2020). It is expected that the Nigerian government would realize COVID-19’s effects on people’s livelihoods could lead to an overnight spike in crime rates, because historically crime rates in Nigeria rise when there is food insecurity occasioned by poverty, food price volatility, disease outbreaks, natural disasters, and violent conflict (Caughron, 2016). While Nigerian social workers could assist in ensuring the welfare of the vulnerable during this pandemic, they have been ignored. This paper assesses the defects of Nigeria’s lockdown measures and explores the role of social workers in cushioning its impact on residents’ livelihood.

## Cushioning the impact of COVID-19 on livelihood

To stem the spread of COVID-19, countries worldwide have employed containment measures such as the washing of hands, social distancing, and lockdowns. International bodies such as the UN Food and Agriculture Organization (FAO), however, recommended that emergency food assistance and social protection programmes should be improved and expanded to help the poor comply with stay-at-home regulations, given that daily income is needed for survival (FAO, 2020). As of March 2020, 283 global programmes are in place, demonstrating a dynamic response to the pandemic and government awareness of the population’s food security concerns (Mengoub, 2020), with governments in the UK, Spain, Germany, Italy, and the US offering social protection packages worth more than 20% of GDP (Krishnan, 2020). Similarly, China, Hong Kong, and Singapore are providing universal one-off cash payments to all citizens (FAO, 2020).

African countries possess neither the wealth nor the technology to be efficient and effective in handling the pandemic like the developed world. It is now evident that poverty in Africa will increase even further, as many of its people will descend into the most vulnerable category after exhausting their savings or being laid off because of the worldwide shutdown of economic activities. This implies that an expanded and increased form of social protection coverage is needed, but this is challenging at present given resource limitations (Vaziralli, 2020). Of the 54 African countries with reported cases, only 22 have introduced fee waivers, seven provide food aid, and five have rolled out cash transfers (Gentilini, 2020). For instance, the Moroccan government has implemented several measures to ensure food security, including giving (in)formal workers who were laid off subsidies of 2,000 MAD (US$195) per month. Similarly, Rwanda, Ghana, Kenya, South Africa, and Uganda have introduced various types of waivers in addition to providing food and cash (Mengoub, 2020; Vaziralli, 2020).

## The defects of Nigeria’s lockdown measures

COVID-19 reached Nigeria when a man who arrived from Italy via Lagos was diagnosed on 27 February 2020 (Nigeria Center for Disease Control (NCDC), 2020). Since then, all 36 states and the federal capital territory have recorded cases. On 30 March 2020, the government announced a complete lockdown, among other containment measures such as social distancing, regular washing of hands, and the use of face masks, in the most affected parts of the country, including Lagos, Ogun, and Abuja (Olarewaju, 2020). Eventually these measures were extended to all parts of Nigeria, including Uyo in Akwa Ibom state, where I (lead author) currently reside. The lockdown has had a tremendous impact on residents.

The lockdown took its toll on me, like everyone, especially with frequent power outages making it impossible to conduct research or engage in certain leisure activities. To minimize my exposure in crowded areas, I went to a remote area to play soccer. I collided with a player and fell badly on the pitch; I thought I was bleeding internally. I could not sleep that night because of severe chest pain. The next day, when my parents went to the market for food, I joined them so I could visit the pharmacy for medicine. We could not take the major roads because of the lockdown, which did not make adequate provision for the sick. The police strictly enforced the lockdown regulation and did not care whether those travelling were ill. We decided to walk along a path in the village and approached an area deliberately blocked by some hoodlums with logs of wood, planks, and worn-out car tires. We had to pay them to pass. We felt helpless. Other people across Nigeria faced the same situations whenever they had to go out for necessities. We were all terrified.

The question is, given Nigeria’s social and economic circumstances, is a strict lockdown the right call? Can it successfully and sustainably defeat the pandemic? As mentioned earlier, countries that have adopted lockdown measures have equally made significant efforts to improve and sustain their residents’ well-being. The Nigerian government failed to consider the deficiencies and the effects of the lockdown it imposed. The impact of the lockdown on people’s livelihoods far outweighs the impact of the virus tearing through Nigeria’s populace, unlike in developed nations, where the opposite holds true (Vaziralli, 2020). Many people have no savings or assets to sustain them during an extended lockdown, and without assistance, most will not survive the lockdown. Regrettably, the government’s interventions to ease the suffering of its people are ridiculous, and it failed to fulfil its promise of unconditional financial assistance for the needy. Non-governmental programmes provided financial and food aid to make up for the insufficiencies of the government’s response. The lack of government assistance has led to a spike in crime rates, as people have turned to crime in sheer desperation to survive. If people could operate businesses as normal without lockdown, people could comfortably meet their daily needs.

In overcrowded ghettos such as Ajengule and Ojuelegba in Lagos, Mararaba in Nasarawa, Etuk and Eka Street in Akwa Ibom, the population density and focus on survival leads to social clustering, not social distancing, rendering the lockdown a waste of time. With the ongoing uncertainty regarding a breakthrough in the search for a vaccine, Nigeria simply does not have the luxury to continue the lockdown. People are frustrated because rent and utility bills have not been waived. The capacity of micro, small, and medium enterprises (MSMEs) and other borrowers to service loans has been diminished, making banks hesitant to grant more loans. Moreover, because of the fall in oil prices during the pandemic, companies have had to revise their budget, which has negatively affected workers’ salaries (Ozili, 2020).

## Exploring the role of Nigeria’s social workers

Social work has always existed in Nigeria, often on the community level. From the precolonial and colonial days to contemporary times, social clubs, missionaries, voluntary agencies, and extended families have provided services and support such as welfare services for children and the elderly, mental healthcare, social planning and development (Okoye, 2013). However, industrialization and globalization have considerably weakened the traditional reliance on extended families for providing welfare assistance, making the need for formal social work practice essential. Despite the high number of social work graduates, President Buhari refused to pass the bill for the professionalization of social work in Nigeria, leaving the hopes of the vulnerable in shambles. Regardless of the pandemic, social welfare programmes in Nigeria are poorly funded, operated by untrained workers and unavailable to those in need, including abandoned children, the homeless, the mentally handicapped, and many others (Ahmed et al., 2017; Ozili, 2020), and in this pandemic, the needs of the vulnerable have been overlooked. Therefore, social workers should be reconsidered and designated as essential workers during and after the pandemic.

In addition, for millions of Nigerians, daily life is a life-and-death struggle as they face numerous challenges on the streets. They can hardly make sense of the lockdown, which places an additional burden on their already difficult lives. The coronavirus is not a death sentence, especially for patients with no chronic ailments, and in the developing world, people are more likely to die of starvation than the virus. Thus, keeping people at home without providing an effective welfare package will undoubtedly result in hunger, anger, frustration, and aggression. Richardson’s (2011) interpretation of the theory of relative deprivation is particularly apt in the current context in Nigeria: the human disposition towards violent tendencies is primarily instigated by frustration. Hence, anger that hinges on frustration, most likely spurred by feelings of being deprived, is more often than not a motivating force for aggressive behaviours that could be manifested as violent crimes (Richardson, 2011). This is evident in the rise of gangsterism and terror groups consisting of young adults in the country.

Nevertheless, the lockdown measures do hold some benefits for Nigeria. The inclusion of social workers can sustainably improve adherence to lockdown regulations, as social workers primarily work with vulnerable groups. Food aid proved impactful in Ecuador, whereas cash payments had a greater impact in Bangladesh, Kenya, and Zambia (Egger et al., 2019; Gentilini, 2020; Handa et al., 2018; Vaziralli, 2020). In Nigeria, both food and cash are required due to the country’s remarkable diversity. Thus, during this pandemic, social workers can help by researching the peculiarities and experiences of vulnerable populations to ensure that food aid and cash transfers that present a viable option for sustenance are distributed to them. Additionally, social workers can help the government to develop culturally appropriate responses to support people and difficult-to-reach communities.

Moreover, some security agencies and other bodies tasked with enforcing the lockdown are using it to their advantage, making curtailing the spread an uphill task. Those who pay bribes are allowed to move about freely, while those who cannot adhere to the lockdown regulations due to hunger completely defy the lockdown, resulting in deadly clashes between citizens and security forces. As of 20 April 2020, the National Human Rights Commission (NHRC) reported 18 deaths caused by security officers and more than 100 complaints received from citizens, especially essential workers, concerning harassment from security officials (BBC News, 2020). If involved, social workers can join forces with the NHRC and journalists to advocate for human rights and condemn the excesses of security officials while providing psychosocial support and counselling to the victims and their families.

With the continuous rise in cases and the need for the lockdown to be relaxed, social workers can partner with the local religious units, authorities and organizations, in mobilizing, educating, and possibly equipping people with the knowledge to take responsibility for their personal safety. Furthermore, social workers could help disseminate information directly to the ghettos and other poor areas where people have no access to TVs, radios, and other mediums through which information is disseminated. This will help people to maintain proper hygiene and use facemasks, as it is impossible to maintain social distancing in these areas. Social workers could make good use of their professional skills and knowledge to identify and contact the most vulnerable families since Nigeria does not have credible demographic data to identify and target the vulnerable. Additionally, social workers should be involved in the psychosocial emergency response planning process for all COVID-19 patients and their families.

From the moment Nigeria recorded its index case to August 2020, the time of writing this article, no information of what social workers can do or are doing to help cushion the impact. Instead, people who are not motivated to provide help on a humanitarian level are the ones tasked with care for the vulnerable.

## Conclusion

Considering the impacts of COVID-19 pandemic revealed in this paper, the government has a duty to ensure that it meets the needs of the people, especially the vulnerable. Who can work more effectively at the grassroots levels than social workers who are generally people oriented? Now is the time for the Nigerian government to rethink its actions and include social workers in its plan to move society forward. Social workers should be considered an essential party in facing the challenges of COVID-19 because of their training and value on helping the vulnerable and human beings.
